# Diagnostic accuracy of dual energy computed tomography for suspected pyogenic spondylodiscitis

**DOI:** 10.1038/s41598-025-04216-9

**Published:** 2025-06-10

**Authors:** Carsten Stelbrink, Paul Jahnke, Friedemann Goehler, Yan Klosterkemper, Matthias Pumberger, Friederike Schömig, Niklas Tuttle, Kerstin Rubarth, Torsten Diekhoff, Julian Pohlan

**Affiliations:** 1 Department of Radiology, Charité - Universitätsmedizin Berlin, Humboldt-Universität zu Berlin, Freie Universität Berlin and Berlin Institute of Health, Charitéplatz 1, 10117 Berlin, Germany; 2 Department of Spine Surgery, Center for Musculoskeletal Surgery, Charité - Universitätsmedizin Berlin, Humboldt-Universität zu Berlin, Freie Universität Berlin, Charitéplatz 1, 10117 Berlin, Germany; 3 Institute of Biometry and Clinical Epidemiology, Charité - Universitätsmedizin Berlin, Humboldt-Universität zu Berlin, Freie Universität Berlin, Charitéplatz 1, 10117 Berlin, Germany; 4 Institute of Medical Informatics, Charité - Universitätsmedizin Berlin, Humboldt- Universität zu Berlin, Freie Universität Berlin, Invalidenstraße 90, 10115 Berlin, Germany; 5 Berlin Institute of Health at Charité (BIH), Anna-Louisa-Karsch-Straße 2, 10178 Berlin, Germany; 6 Johnson&Johnson Innovative Medicine, Janssen-Cilag GmbH, Johnson&Johnson Platz 1, 41470 Neuss, Germany

**Keywords:** Spine, Spondylodiscitis, Degeneration, Dual-energy computed tomography, CMap, Magnetic resonance imaging, Bacterial infection, Computed tomography, Magnetic resonance imaging, Skeleton

## Abstract

While magnetic resonance imaging (MRI) is the diagnostic method of choice, we here analyze the diagnostic potential of dual-energy computed tomography (DECT) in differentiating abnormal discs from normal-appearing discs and in differentiating between infectious and degenerative conditions. Twenty-eight patients with suspected spondylodiscitis who underwent DECT and MRI of the spine were retrospectively included. Eighteen patients were diagnosed with spondylodiscitis and ten patients with degenerative disc disease. A combined clinical reference standard for the diagnosis was used. One abnormal disc and one normal-appearing disc per patient were included. Three blinded readers analyzed CT, DECT cMaps and MRI images. Quantitative analysis was performed in standardized regions of interest placed in each of the two discs included. Mixed-model analysis was used to identify correlations between CT density alterations and spondylodiscitis or degenerative disc disease. Diagnostic accuracy for differentiating abnormal discs and normal-appearing discs: 69.7% (95% CI, 56.0 to 81.2) for CT, 76.8% (95% CI, 63.6 to 87.0) for CT + DECT, 58.9% (95% CI, 45.0 to 71.9) for MRI; for differentiating spondylodiscitis and degenerative disc disease: 64.3% (95% CI, 44.1 to 81.4) for CT, 60.7% (95% CI, 40.6 to 78.5) for CT + DECT, 53.6% (95% CI, 33.9 to 72.5) for MRI. Mixed-model analysis revealed that normal-appearing discs had higher average density than abnormal discs in DECT (mean difference = 47.0 HU (95% CI, 32.8 to 61.3), p = < 0.001). In summary, both qualitative and quantitative DECT can distinguish normal-appearing discs from abnormal discs. Spondylodiscitis and degenerative disc disease were not distinguished accurately by DECT in this study.

**Supplementary Information:**

The online version contains supplementary material available at 10.1038/s41598-025-04216-9.

## Introduction

Spondylodiscitis (SD) is a spinal infection of the intervertebral disc (IVD) and adjacent vertebral bodies^1,2^. While the incidence is on the rise^3–5^, delayed diagnosis is not uncommon and may lead to high morbidity and even death^6–11^. Well-established risk factors are higher age, chronic diseases, immunosuppressive treatment, and intravenous drug abuse^5,8,12–14^. The most common infectious pathway is hematogenous dissemination in patients with infections of the skin, soft tissue, respiratory, or urogenital system^15^. While several pathogens are known to cause SD, *Staphylococcus aureus* has the highest prevalence^2,5,16,17^. The diagnosis of SD is based on clinical, microbiological, and radiological findings. The clinical presentation of SD may be vague with initially unspecific symptoms like back pain or fever, and general weakness^15,16^.

Magnetic resonance imaging (MRI) is the imaging gold standard for establishing the diagnosis of SD, providing high sensitivity and specificity of 96% and 92%, respectively, for detecting spinal infection^18,19^. However, MRI might not be readily available in certain regions or hospitals or might be precluded by contraindications such as severe claustrophobia or unsafe metallic objects^20,21^.

Computed tomography (CT) is more widely available and will often be the first imaging modality in patients with severe illness and unclear infectious focus. Despite the use of ionizing radiation, it has hardly any contraindications. Dual-energy computed tomography (DECT) is an established imaging modality first used in urology and rheumatology^22,23^. Using the so called virtual-non calcium (VNCa) DECT has been investigated to detect bone marrow lesions^24,25^. By applying three-material decomposition algorithm, DECT provides a collagen-sensitive imaging modality by generating collagen-sensitive maps (cMaps)^26,27^ which may also facilitate imaging of collagen-rich structures such as ligaments and tendons^25,28,29^. Because of the high content of collagen and chondroitin sulfate in human IVDs DECT cMaps are also capable to show alterations microstructural changes in it^25,30^. Foti et al.^31^ report that DECT can also be used to identify osseous and soft tissue alterations in nontraumatic settings such as infection.

Therefore, the purpose of this study is to analyze the capability of DECT cMaps in differentiating abnormal IVDs from normal-appearing discs and in differentiating SD from degenerative disc disease (DDD) as the underlying cause in patients with suspected SD.

## Methods

### Ethics approval

This study was approved by the local ethics committee, Campus Charité Mitte, Charitéplatz 1, 10,117 Berlin, under EA1/230/19. The Declaration of Helsinki was respected. Written informed patient consent was not required because of its retrospective design. All research was performed in accordance with relevant guidelines and regulations.

### Patient recruitment

We searched the radiology information system (RIS) of our department to retrospectively identify patients. Inclusion criteria were: Patients with suspected SD of the lumbar or thoracic spine who underwent DECT for diagnosis or for biopsy guidance and MRI within four weeks of the DECT scan from April 2014 to July 2021. The reference standard for the diagnosis of either SD or DDD was established taking the clinical presentation, imaging findings, microbiological results, and the final diagnosis at discharge into account. Patients with unavailable images or insufficient image quality in MRI or DECT were excluded.

### Inclusion and exclusion criteria of patient IVDs

**Fig. 1:**
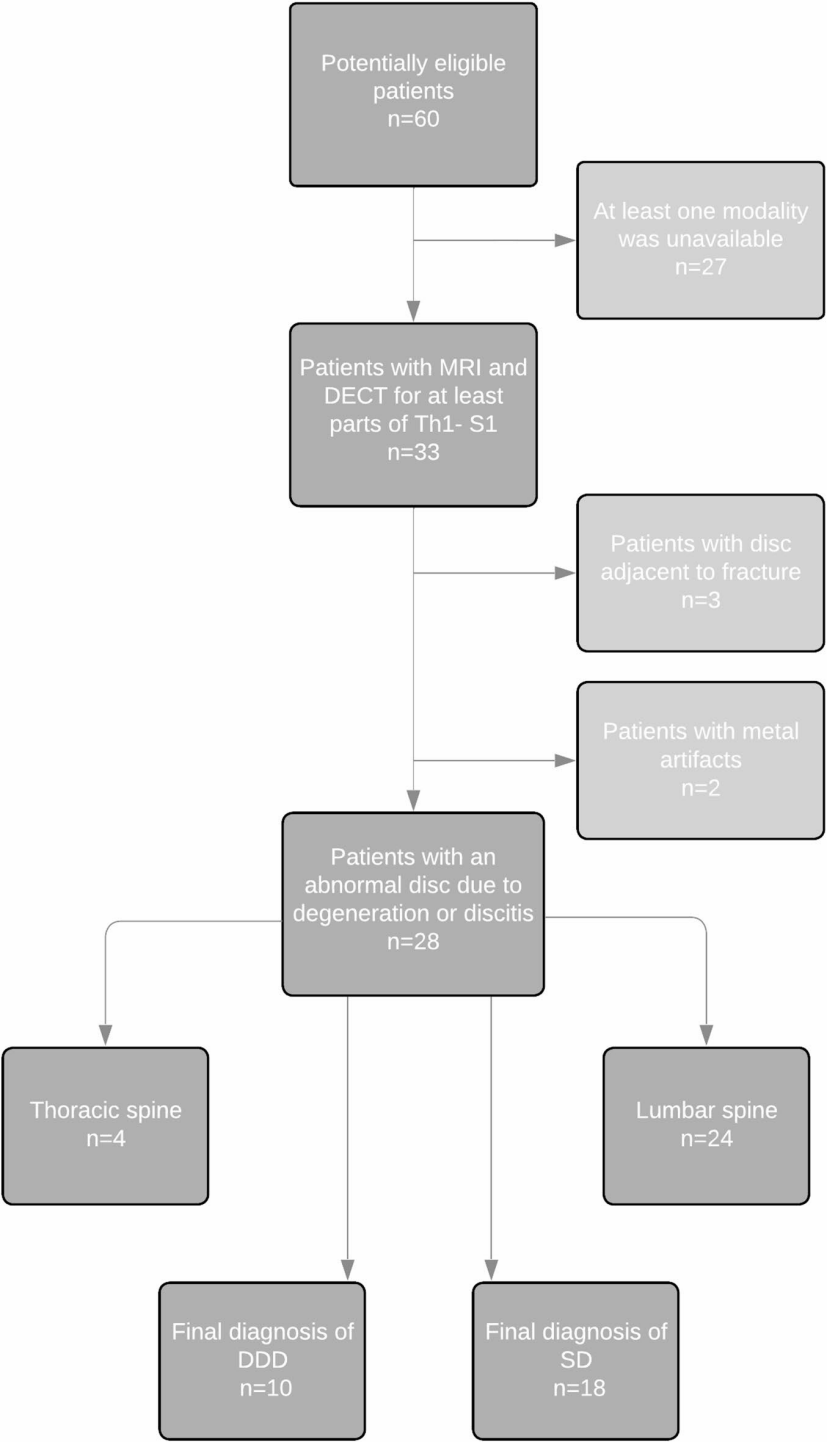
Flow chart of patient selection. Patients with suspected SD of the thoracic or lumbar spine who underwent both DECT and MRI between 2014 and 2021 were retrospectively identified. Reasons for exclusion during patient recruitment are provided.DECT: Dual-energy computed tomography; DDD: degenerative disc disease; SD: spondylodiscitis

Two discs were analyzed per patient: the abnormal disc and one normal-appearing disc. The two discs included per patient were required to be depicted by both MRI and DECT cMaps in the period mentioned above *(*Fig. 1*)*.

Patients with abnormal discs or normal-appearing discs adjacent to vertebral fractures or severe artifacts (e.g., due to metal implants) were excluded. A minimum distance of at least two levels between the abnormal disc and normal-appearing disc was applied, for example L2/3 (normal) and L4/5 (abnormal), to avoid a pathological affection of the respective IVD analyzed.

### DECT protocol

DECT images were acquired on a single-source DECT scanner (Aquilion ONE Vision, Canon Medical Systems) equipped with a 320-row detector to allow repeated scanning without table movement. Dual-energy datasets were obtained by sequential acquisition of two volumes at 135 kVp and 80 kVp. Rotation time was 0.275 s with a changeover time of 0.5 s between acquisitions. Exposure control was set to a standard deviation of 12. Z-axis coverage was extended by enabling the wide-volume mode for all scans exceeding 16 cm^32^.

#### Postprocessing

Dual-energy datasets were reconstructed with 0.5 mm slice thickness using a medium soft tissue kernel without beam hardening compensation and image-based iterative reconstruction (AIDR-3D, standard level). Water-iodine material maps were generated in addition to the conventional gray-scale images to be further used for DECT reconstruction. The datasets were then further postprocessed at the CT console (Dual-Energy Raw Data Analysis Version 6.0) to generate collagen/chondroitin maps (cMaps) using a collagen-specific gradient of 1.1 and applying a three-material-decomposition algorithm.

### MRI protocol

Each MRI was performed using a clinical 1.5-T standard imager (MAGNETOM Avanto; Siemens Healthineers or MAGNETOM Symphony Vision; Siemens Healthineers) and included both the T1-weighted (repetition time, 551 ms; echo time, 12 ms; scan time, 5 min 12 s) sequence and a short tau inversion recovery (STIR) sequence (repetition time, 6150 ms; echo time, 31 ms; inversion time, 150 ms; scan time, 4 min 15 s) at a slice thickness of 3 mm. Contrast agent was administered based on clinical indication, the primary diagnostic question, and contraindications.

### Image reading

For the reading, the images were uploaded into an in-house developed software tool for blinded assessment^33^. Three readers scored the randomized image datasets of 28 patients in coronal, axial, and sagittal reconstructions of conventional CT, CT + DECT cMaps and MRI. The readers were blinded to all but the included discs, the clinical characteristics, the final diagnosis, and the results of the other imaging modalities when scoring the images. The following main features were rated: (1) the general impression whether a normal-appearing disc, DDD or SD was presented (2) the diagnostic confidence. The following additional features were rated: (1) destruction of endplates, (2) lysis of endplates, (3) sclerosis of endplates, (4) vertebral fractures, (5) spinal alignment, (6) pre-/paravertebral abscess, (7) vacuum phenomena, and (8) disc height. MR images were additionally rated for presence of the following findings: (9) bone marrow substitution, (10) bone marrow edema, 11) fluid-in-disc sign, 12) degeneration, 13) intradiscal abscess, 14) epidural abscess, and 15) soft tissue abscess. Reader 1 had 14 years of experience (musculoskeletal radiologist), reader 2 had nine years of experience (radiologist), and reader 3 had three and a half years of experience (orthopedic surgeon). A scoring finding was considered positive when at least two readers agreed on its presence. To reduce recall bias, the interval between the reading sessions for each modality was at least three days.

### Quantitative analysis

A total of 56 IVDs from 28 patients were analyzed quantitatively. Standardized regions of interest (ROIs) of 20 mm^2^ were placed in the anulus fibrosus (AF), and the nucleus pulposus (NP) and, if present, in the inflammatory lesion (IL) in oblique axial reformations of 3 mm slice thickness both in 135 kVp CT images and in DECT cMaps.

### Statistics

Densities in Hounsfield units (HU) measured in the ROIs were analyzed descriptively using appropriate summary statistics such as the mean and standard deviation for metric variables or absolute and relative frequencies for categorical data. Because of the clustered structure of the data, mixed-model analysis was performed using the following fixed factors: (1) final diagnosis, (2) gender, (3) level of abnormal disc, (4) ROI localization, (5) patient age, and (6) identifier of abnormal disc. The subject identifier was used as a random factor, and covariance structure was modeled by variance components. A possible effect of ROI localization on IVD density in HU was investigated using an interaction term containing the patient’s final diagnosis and the ROI localization within the disc. P-values and confidence intervals were not adjusted for multiplicity and are therefore interpreted in an exploratory, hypothesis-generating manner. Diagnostic accuracy parameters were calculated from the results of the three readers assessing the general impression whether a normal-appearing disc, DDD or SD was presented. According to these results, Fleiss kappa was calculated for interrater reliability. All data were collected in Excel tables (Microsoft Excel for Mac Version 16.52) for further analysis. Statistical analysis was performed using SPSS version 26.0 (IBM Corp., Armonk, NY, USA). A p-value < 0.05 was considered significant. GraphPad Prism (Version 8) was used for data visualization.

## Results

### Characteristics of the study population

Mean age was 62.4 ± 18.5 standard deviation (sd) years in the DDD group and 65.4 ± 15.2 sd years in the SD group. The proportion of female patients was 80.0% (*n* = 8) in the DDD group and 27.8% (*n* = 5) in the SD group. In the SD group, 22.2% (*n* = 4) of the patients had involvement of the thoracic spine, and 77.8% (*n* = 14) involvement of the lumbar spine. All ten patients in the DDD group had lumbar spine involvement (100%). Blood cultures were positive in 33.3% of patients (*n* = 6) in the SD group. Ten patients of the SD group underwent CT-guided biopsy (positive in four patients, 40.0%; Supplementary Table 1).

### Diagnostic accuracy and interrater reliability

Diagnostic accuracy for differentiation between abnormal discs and normal-appearing discs was 69.7% (95% CI, 56.0 to 81.2) for conventional CT images, 76.8% (95% CI, 63.6 to 87.0) for CT + DECT cMaps, and 58.9% (95% CI, 45.0 to 71.9) for MRI. Diagnostic accuracy for differentiation between SD and DDD was 64.3% (95% CI, 44.1 to 81.4) for conventional CT images, 60.7% (95% CI, 40.6 to 78.5) for CT + DECT cMaps, and 53.6% (95% CI, 33.9 to 72.5) for MRI images. The accuracy results are summarized in Table 1.

**Table 1:** Accuracy results of the three raters with different levels of experience for the three sets of images analyzed. The first row presents accuracy data for the differentiation between ADs and NADs. The second row shows the accuracy data for the differentiation between ADs in the SD group and ADs in the DDD group.

	CT	CT+ DECT cMap	MRI
	% 95%CI	% 95%CI	% 95%CI
AD vs. NAD			
Sensitivity	89.3(71.8 to 97.7)	85.7 (67.3 to 96.0)	89.3 (71.8 to 97.7)
Specificity	50.0 (30.6 to 69.3)	67.9 (47.6 to 84.1)	28.6 (13.2 to 48.7)
DA	69.7 (56.0 to 81.2)	76.8 (63.6 to 87.0)	58.9 (45.0 to 71.9)
Positive LR	1.8 (1.2 to 2.6)	2.7 (1.5 to 4.7)	1.2 (1.0 to 1.6)
Negative LR	0.2 (0.1 to 0.7)	0.2 (0.1 to 0.5)	0.4 (0.1 to 1.3)
SD vs. DDD			
Sensitivity	61.1 (35.7 to 82.7)	77.8 (52.4 to 93.6)	83.3 (58.6 to 96.4)
Specificity	70.0 (34.7 to 93.3)	30.0 (6.7 to 65.2)	0.0 (0.0 to 30.8)
DA	64.3 (44.1 to 81.4)	60.7 (40.6 to 78.5)	53.6 (33.9 to 72.5)
Positive LR	2.0 (0.7 to 5.6)	1.1 (0.7 to 1.8)	0.8 (0.7 to 1.0)
Negative LR	0.6 (0.3 to 1.1)	0.7 (0.2 to 2.7)	0.0 (0.0 to 0.0)

*CT: Computed tomography; DA: Diagnostic accuracy; DDD: Degenerative disc disease; DECT: Dual-energy computed tomography; LR: Likelihood ratio; MRI: Magnetic resonance imaging; NAD: Normal-appearing disc; SD: Spondylodiscitis*

The distribution of scoring results for each of the three imaging modalities is displayed in a contingency table (*Supplementary Table 2*). Analysis of interrater reliability yielded moderate agreement with a Fleiss K of 0.475 (95% CI, 0.472 to 0.479) for the scoring of CT images and moderate agreement with a Fleiss K of 0.432 (95% CI, 0.428 to 0.435) for CT + DECT cMaps. Interrater reliability for MRI yielded fair agreement with a Fleiss K of 0.333 (95% CI, 0.329 to 0.336). Diagnostic confidence ratings are shown separately for each reader in a table (*Supplementary Table 3).* Results of the feature reading are compiled for each imaging modality and IVD condition (*Supplementary Table 4)*.

### Quantitative analysis of disc density

**Fig. 2:**
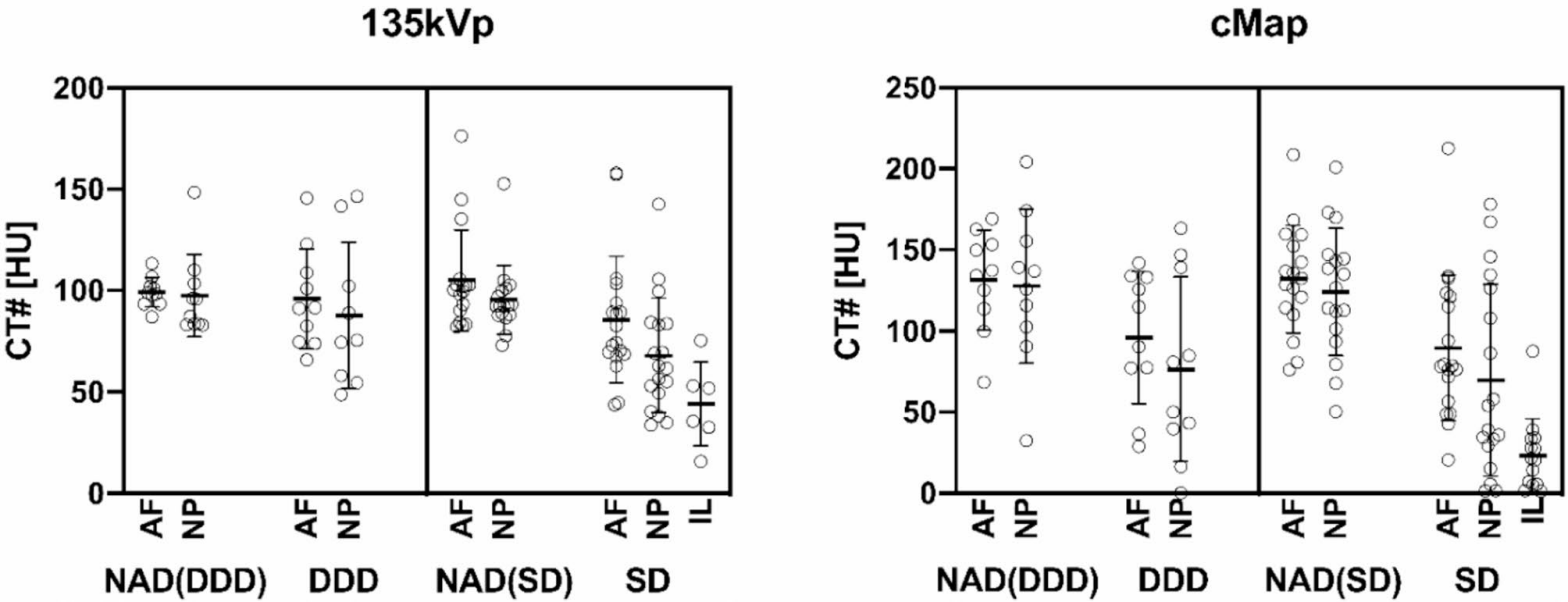
Quantitative disc analysis in CT 135 kVp images and in DECT cMap reconstructions. NADs are compared with ADs separately for the DDD group and the SD group. The plots are based on the data reported in the results section. DDD group: NAD: 99.1 ± 7.3 SD HU (AF) vs. 97.5 ± 20.3 SD HU (NP) in 135 kVp; AD: 95.8 ± 24.6 SD HU (AF) vs. 87.8 + 36.0 SD HU (NP) SD group: NAD: 105.0 ± 25.0 SD HU (AF) vs. 95.5 ± 16.9SD HU (NP); AD: 85.8 ± 31.2 SD HU (AF) vs. 68.0 ± 28.3 SD HU (NP); ILs in ADs (44.1 ± 20.7 SD HU (IL) vs. 67.9 ± 28.2 SD HU (NP) 135kVp. NADs with *n* = 28; DDD with *n* = 10; SD with *n* = 18. *AF: Anulus fibrosus; cMap*: Collagen-/chondroitin-sensitive map; *DDD: Degenerative disc disease; IL: Inflammatory lesion; NAD: Normal-appearing disc; NP: Nucleus pulposus; AD: Abnormal disc; SD: Spondylodiscitis; HU: Hounsfield units*.

In DECT cMaps, normal-appearing discs had higher density than the corresponding abnormal discs in the same patients in the DDD group (131.3 ± 30.9 sd HU (AF); 127.6 ± 47.4 sd HU (NP) vs. 95.9 ± 40.7 sd HU (AF); 76.4 ± 56.9 sd HU (NP). In the SD group, normal-appearing discs also had higher density than the corresponding abnormal discs (132.0 ± 33.1 sd HU (AF); 124.1 ± 39.1 sd HU (NP) vs. 89.5 ± 44.8 sd HU (AF); 69.6 ± 59.0 sd HU (NP) in DECT cMaps). ILs in abnormal discs of the SD group had a lower average density than the NP of the corresponding abnormal discs (23.3 ± 22.6 sd HU (IL) vs. 69.6 ± 59.0 sd HU (NP) in DECT cMaps). Plots and the results of the 135 kVp CT images measurement are presented in Fig. 2.

Mixed-model analysis revealed a statistically significant difference in IVD density between normal-appearing discs and abnormal discs regardless of the final diagnosis: normal-appearing discs had a higher average density compared to abnormal discs both in 135 kVp CT images and in DECT cMaps (mean difference = 17.4 HU (95% CI, 10.2 to 24.6), *p* < 0.001) vs. mean difference = 47.0 HU (95% CI, 32.8 to 61.3), *p* < 0.001).

Furthermore, mixed-model analysis revealed a difference in IVD density between the DDD and SD groups with the magnitude of the difference varying with the localization of the ROIs both in 135 kVp CT images and in DECT cMaps. The density of the AF was higher than that of the NP in 135 kVp CT images in the DDD group (4.9 HU (95% CI, −6.8 to 16.7), *p* = 0.41) and lower in DECT cMaps in the DDD group (11.6 HU (95% CI, −11.6 to 34.8), *p* = 0.33). In the SD group, the density of the AF was higher than that of the NP both in 135 kVp CT images and in DECT cMaps (13.7 HU (95% CI, 4.9 to 22.6), *p* = 0.003 vs. 14.1 HU (95% CI, −3.7 to 31.8), *p* = 0.12).

Density of ILs in abnormal discs of the SD group was below that of the AF and the NP of the corresponding abnormal discs both in 135 kVp CT images and in DECT cMaps (−8.3 HU (95% CI, −29.7 to 13.1), *p* = 0.99 (AF) and − 22.0 HU (95% CI, −39.7 to −4.4), *p* = 0.02 (NP) vs. −34.4 HU (95% CI, −65.1 to 31.9), *p* = 1 (AF) and − 48.5 HU (95% CI, −73.2 to −23.7), p = < 0.001 (NP)). Additional results of the confounder analysis are compiled in *supplementary Table 5*.

## Discussion

Our study analyzed for the first time the diagnostic accuracy of DECT cMaps in the context of inflammation at the spine. CT, DECT cMaps, and MRI had reasonably high sensitivity in identifying abnormal discs in SD and DDD and differentiating them from normal-appearing discs in the same patients with CT + DECT cMaps showing the highest specificity for this task. Nonetheless, no imaging modality alone was suited to decide whether DDD or SD was present. Regarding the small sample size, a superiority of DECT imaging within the image reading in this study can still not be postulated. This is also reflected by the results of our quantitative analysis of the DECT data, which showed less overall difference between degeneration and infection. However, we found the nucleus pulposus to be more markedly affected in infection and the anulus fibrosus in degeneration, which may provide a clue for better interpretation of DECT cMaps in the future. Appropriately, our results show an additional statistically significantly decrease in density of ILs compared to the NPs of the abnormal discs in patients with SD. However, differentiating between SD an DDD remains a challenge.

**Fig. 3:**
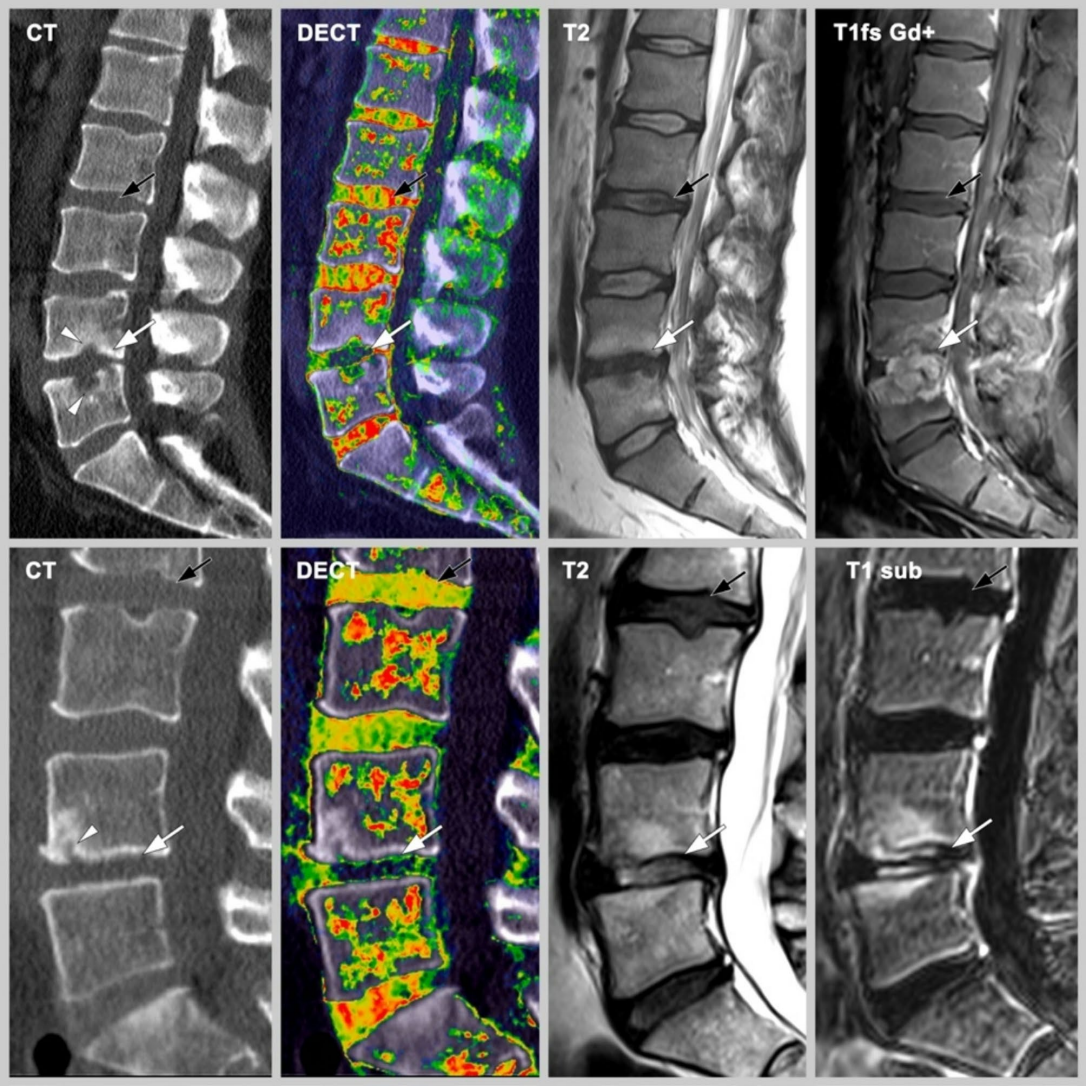
Images obtained in two patients with different spinal abnormalities. Images are sagittal reformations at 3 mm slice thickness and include conventional CT images, DECT cMaps, and T2-weighted MR images, and T1-weighted MR images T1fs Gd + and T1 sub. Patient 1 (upper row): Spondylodiscitis of the L4/5 disc (white arrow). Patient 2 (lower row): Disc degeneration (white arrow) with erosion (white arrowhead) at the L4/5 level. The black arrows point to normal discs for comparison, respectively.*CT: Computed tomography; cMaps*: Collagen-/chondroitin-sensitive map; *DECT: Dual-energy computed tomography; MRI: Magnetic resonance imaging*.

Booz et al. used virtual non-calcium DECT for detection of lumbar disc herniation in comparison with standard gray-scale CT^35^. Differently, Schoemig et al. used DECT cMaps to describe IVD morphology und showed excellent correlation between DECT and MRI in disc herniation^32^. However, in an analysis of our own group, Pohlan et al. showed in an phantom model that DECT cMaps can detect different collagen concentrations in agar solution by measuring concentration-dependent HU and add to the existing knowledge, that the signal detected by DECT cMaps is not only due to collagen, but also to chondroitin sulfate, the ladder of which had a very strong signal^36^. Showing different HU densities in conventional CT and CT + DECT cMap suggest a more selective imaging of collagen and chondroitin sulfate which Pohlan et al. showed in recent studies^30^. Additionally, we have demonstrated that IVD density is lower in elderly patients^30^ while the present study shows no statistically significant correlation between age and IVD density. However, our results suggest that there is a correlation between the density and morphological changes of IVDs in comparison to normal-appearing discs in the same patients, which is consistent with previous findings. Also, we have shown that DECT cMaps allow detection of inflammatory lesions in the NP in an ex vivo biophantom model^36^. Reliable lesion identification is important to improve the yield of CT-guided biopsies given the reported success rates of 30–91%^37–39^, which is consistent with a 40% success rate of biopsies (four of ten patients). In our population, levels of spinal involvement are consistent with the distribution reported in literature for both DDD and SD^40–42^. Patients with SD in our study had a mean age of about 65 years, confirming that higher age is an important risk factor for SD^13,14,41^. The proportion of female patients was 80.0% in the DDD group and 27.8% in the SD group. There is no evidence for sex-specific differences in the occurrence of these two spinal conditions in literature. Blood cultures were positive in six patients with SD (33.3%), which is similar to published rates^38^. Furthermore, our results show that CT + DECT in combination allow highly accurate identification of disc pathologies in general which is consistent to previous findings which showed that CT + DECT is superior against CT only^43^ and also differentiation between degenerative conditions and inflammatory SD lesions. Images of one each exemplary patient per pathology are shown in Fig. 3.

However, differentiation of these pathologies is more difficult using qualitative assessment alone. Importantly, while there are many sequences and better soft tissue contrast using MRI, DECT cMaps may be simpler to interpretate. In addition, not all patients received contrast-enhanced MRI in the clinical routine. This may also explain the lower diagnostic accuracy of MRI in this study. However, Foti et al. described a non-significant drop in diagnostic performance by evaluating native MRI against contrast-enhanced MRI according to spondylodiscitis^34^.

Our study has several limitations. Only 28 patients were included in this exploratory study, which may explain why some differences did not reach statistical significance. Therefore, our results need to be confirmed in a larger study population. Mean age was over 60 years in both groups. However, previous studies found that ageing and age-related spinal degeneration reduce the collagen and chondroitin content of intervertebral discs measured by DECT cMaps. Contrast between normal-appearing and abnormal discs should even be better in younger patients. Thus, our results cannot be transferred easily in younger populations. Due to radiation protection, there is no matched control cohort in our study so normal-appearing and abnormal discs has been included in the same patient respectively. Due to the retrospective nature of our study, not all patients underwent contrast injection in MRI and it is difficult to assess certain clinical parameters that would be available in a prospective study. The reference standard whether SD or DDD was diagnosed is based on the combination of clinical, microbiological and radiological findings. Not all patients of the SD group got germ detection in blood cultures or in biopsy as a definite diagnosis. However, this stands in line with published findings. It is still unclear which groups of patients may benefit most from a primary or additional DECT examination. We were only able to include patients up to the year 2021. Therefore, the transfer of our results to newer scanners might be limited. Using the three-material decomposition is basically possible on every CT scanner while postprocessing may not always be easily available. Until now, results are only published on a single vendor. Results may not be easily transferred when using software of different vendors. However, this question should be targeted in future studies.

Our findings suggest that both qualitative and quantitative DECT can distinguish normal-appearing from abnormally altered discs. While neither qualitative nor quantitative DECT allows reliable differentiation of inflamed and degenerated discs, DECT cMaps are particularly useful in identifying inflammatory lesions within intervertebral discs affected by SD. Overall, our results are consistent with previous findings by our group and other investigators and are relevant to increase the value of DECT cMaps in imaging IVD pathology. Larger prospective studies with bigger sample sizes and multicenter setting are needed to confirm these results.

## Electronic supplementary material

Below is the link to the electronic supplementary material.

Supplementary Material 1

## Data Availability

Data and materials that were generated and analyzed during this study are available from CS. The data are provided within the manuscript or in the supplementary information files.
