# Supplementary material for: Diagnostic accuracy of dual energy computed tomography for suspected pyogenic spondylodiscitis
Source: Sci Rep. 2025 Jun 10;15:20040. doi: 10.1038/s41598-025-04216-9 (PMC12152176; doi:10.1038/s41598-025-04216-9)
Supplement: Supplementary file 1 — Supplementary Material 1 [file 41598_2025_4216_MOESM1_ESM.docx]

**Title**

**Diagnostic accuracy of dual energy computed tomography for suspected pyogenic spondylodiscitis**

**Authors**

Carsten Stelbrink­ ^1^, Paul Jahnke ^1^ Friedemann Goehler ^1^, Yan Klosterkemper ^1^, Matthias Pumberger ^2^, Friederike Schömig ^2^, Niklas Tuttle ^2^, Kerstin Rubarth ^3,4^, Torsten Diekhoff ^1 *^, Julian Pohlan ^1,5,6 *^

**Affiliation**

1Department of Radiology, Charité - Universitätsmedizin Berlin, Humboldt-Universität zu Berlin, Freie Universität Berlin, Charitéplatz 1, 10117, Berlin, Germany

2Department of Spine Surgery, Center for Musculoskeletal Surgery, Charité - Universitätsmedizin Berlin, Humboldt-Universität zu Berlin, Freie Universität Berlin, Charitéplatz 1, 10117, Berlin, Germany

3Institute of Biometry and Clinical Epidemiology, Charité - Universitätsmedizin Berlin, Humboldt-Universität zu Berlin, Freie Universität Berlin, Charitéplatz 1, 10117, Berlin, Germany

4Institute of Medical Informatics, Charité - Universitätsmedizin Berlin, Humboldt-Universität zu Berlin, Freie Universität Berlin, Invalidenstraße 90, 10115, Berlin, Germany

5Berlin Institute of Health at Charité (BIH), Anna-Louisa-Karsch-Straße 2, 10178, Berlin, Germany

6Johnson&Johnson Innovative Medicine, Janssen-Cilag GmbH, Johnson&Johnson Platz 1, 41470, Neuss, Germany

*shared senior authors

**Corresponding author**

Carsten Stelbrink, Charité - Universitätsmedizin Berlin, Luisenstrasse 7, Berlin 10117, Germany.
E-Mail: carsten.stelbrink@charite.de

**Supplementary data**

Supplementary table 1: Results for patients with CT-guided biopsies, blood cultures and other findings in the SD group including demographic and clinical information.

*BC: Blood culture; CRP: C-reactive protein; F: Female; HP: histopathological finding; L: Lumbar spine segment; LK: Leukocytes; M: Male; Th: Thoracic spine segment; +: Positive yield; -: Negative yield*

|  | **Age** | **Sex** | **Disc level** | **Biopsy** | **Blood culture** | **CRP; LK; other** |
| --- | --- | --- | --- | --- | --- | --- |
| **1** | 33 | M | Th11/Th12 | + | - | 39.7mg/l; 7.5/nl |
| **2** | 38 | M | L1/L2 | - | - | 188.7mg/l; 8.1/nl |
| **3** | 60 | M | L4/L5 | - | - | 30.0mg/l; 6.8/nl |
| **4** | 70 | M | L4/L5 | - | - | 0.7mg/l; 5.6/nl; HP |
| **5** | 72 | F | Th9/Th10 | + | + | 64.9mg/l; 13.9/nl |
| **6** | 74 | M | L1/L2 | - | + | 90.6mg/l; 5.6/nl |
| **7** | 81 | M | L2/L3 | - | + | 65.3mg/l; 7.2/nl; |
| **8** | 83 | M | L2/L3 | + | - | 48.6mg/l; 6.7/nl |
| **9** | 83 | M | L3/L4 | + | - | 260.1mg/l; 7.6/nl |
| **10** | 87 | F | L1/L2 | - | + | 21.8mg/l; 4.5/nl |

Supplementary table 2: Contingency table results for the ratings of three raters with different levels of experience. The upper portion of the table presents the results for the differentiation between abnormal and normal-appearing discs for the three sets of images analyzed in this study. The second row shows the results for the differentiation between abnormal discs in the SD group and abnormal discs in the DDD group for the three sets of images analyzed.

*AD: abnormal disc; CT: Computed tomography; DDD: Degenerative disc disease; DECT+: Dual-energy computed tomography cMaps in combination with conventional CT; MRI: Magnetic resonance imaging; NAD: Normal-appearing disc; SD: Spondylodiscitis*

|  | **ADs** | **NADs** | **Total** |  | **ADs** | **NADs** | **Total** |  | **ADs** | **NADs** | **Total** |
| --- | --- | --- | --- | --- | --- | --- | --- | --- | --- | --- | --- |
| **CT(AD)** | 25 | 14 | 39 | **DECT+(AD)** | 24 | 9 | 33 | **MRI(AD)** | 25 | 20 | 45 |
| **CT(NAD)** | 3 | 14 | 17 | **DECT+(NAD)** | 4 | 19 | 23 | **MRI(NAD)** | 3 | 8 | 11 |
| **Total** | 28 | 28 | 56 | **Total** | 28 | 28 | 56 | **Total** | 28 | 28 | 56 |
|  | **SD** | **DDD** | **Total** |  | **SD** | **DDD** | **Total** |  | **SD** | **DDD** | **Total** |
| **CT(SD)** | 11 | 3 | 14 | **DECT+(SD)** | 14 | 7 | 21 | **MRI(SD)** | 15 | 10 | 25 |
| **CT(DDD)** | 7 | 7 | 14 | **DECT+(DDD)** | 4 | 3 | 7 | **MRI(DDD)** | 3 | 0 | 3 |
| **Total** | 18 | 10 | 28 | **Total** | 18 | 10 | 28 | **Total** | 18 | 19 | 28 |

Supplementary table 3: Mean diagnostic confidence values for the three raters separately for each of the three imaging modalities, independent of whether the decision was right or wrong.

*CT: Computed tomography; cMaps:* collagen-/chondroitin-sensitive map; *DECT: Dual-energy computed tomography; MRI: Magnetic resonance imaging*

|  | **Reader 1** | **Reader 2** | **Reader 3** |
| --- | --- | --- | --- |
| **Conventional CT** | 7.9 | 6.6 | 8.0 |
| **CT+DECT cMaps** | 8.8 | 6.2 | 8.1 |
| **MRI** | 8.0 | 6.2 | 7.6 |

Supplementary table 4: For the scoring of additional features, agreement of at least two readers was considered a positive finding. The readers were blinded to all clinical characteristics, the final diagnosis, and the results of the other imaging modalities at the time of scoring. The results are percentages for each feature in the imaging modalities respectively for ADs of SD, DDD, and for NADs.

*AD: Abnormal disc; CT: Computed tomography; DECT+: Dual-energy computed tomography cMaps in combination with conventional CT; MRI: Magnetic resonance imaging; NAD: Normal-appearing disc; DDD: Degenerative disc disease; SD: Spondylodiscitis; -: Features only assessable on MRI*

|  |  | **CT** |  |  | **DECT+** |  |  | **MRI** |  |  |
| --- | --- | --- | --- | --- | --- | --- | --- | --- | --- | --- |
|  |  | **NAD** | **DDD** | **SD** | **NAD** | **DDD** | **SD** | **NAD** | **DDD** | **SD** |
| **1** | **Destruction of endplates** | 7.1% (2/28) | 60.0% (6/10) | 88.8% (16/18) | 7.1% (2/28) | 60.0% (6/10) | 83.3% (15/18) | 7.1% (2/28) | 80.0% (8/10) | 61.1% (11/18) |
| **2** | **Lysis of endplates** | 10.7% (3/28) | 20.0% (2/10) | 61.1% (11/18) | 0.0% (0/28) | 10% (1/10) | 66.6% (12/18) | 0.0% (0/28) | 10.0% (1/10) | 22.2% (4/18) |
| **3** | **Sclerosis of endplates** | 28.6% (8/28) | 70.0% (7/10) | 33.3% (6/18) | 10.7% (3/28) | 50.0% (5/10) | 33.3% (6/18) | 7.1% (2/28) | 20.0% (2/10) | 11.1% (2/18) |
| **4** | **Vertebral fractures** | 0.0% (0/28) | 10.0% (1/10) | 27.8% (5/18) | 0.0% (0/28) | 20.0% (2/10) | 38.9% (7/18) | 0.0% (0/28) | 40.0% (4/10) | 16.7% (3/18) |
| **5** | **Spinal alignment** | 0.0% (0/28) | 20.0% (2/10) | 27.8% (5/18) | 0.0% (0/28) | 10.0% (1/10) | 11.1% (2/18) | 0.0% (0/28) | 10.0% (1/10) | 16.7% (3/18) |
| **6** | **Pre-/paravertebral abscess** | 0% (0/28) | 0.0% (0/10) | 11.1% (2/18) | 0.0% (0/28) | 0.0% (0/10) | 38.8% (7/18) | 0.0% (0/28) | 0.0% (0/10) | 22.2% (4/18) |
| **7** | **Vacuum phenomena** | 3.6% (1/28) | 20.0% (2/10) | 5.5% (1/18) | 0.0% (0/28) | 20.0% (2/10) | 5.5% (1/18) | 0.0% (0/28) | 0.0% (0/10) | 0.0% (0/18) |
| **8** | **Disc height** | 39.3% (11/28) | 50.0% (5/10) | 88.8% (16/18) | 14.2% (4/28) | 60.0% (6/10) | 77.7% (14/18) | 39.3% (11/28) | 60.0% (6/10) | 77.8% (14/18) |
| **9** | **Bone marrow substitution** | - | - | - | - | - | - | 7.1% (2/28) | 10.0% (1/10) | 50.0% (9/18) |
| **10** | **Bone marrow edema** | - | - | - | - | - | - | 3.6% (1/28) | 60.0% (6/10) | 55.5% (10/18) |
| **11** | **fFuid-in-disc sign** | - | - | - | - | - | - | 7.1% (2/28) | 10.0% (1/10) | 77.8% (14/18) |
| **12** | **Degeneration** | - | - | - | - | - | - | 53.6% (15/28) | 70% (7/10) | 66.7% (12/18) |
| **13** | **Intradiscal abscess** | - | - | - | - | - | - | 3.6% (1/28) | 0.0% (0/10) | 38.8% (7/18) |
| **14** | **Epidural abscess** | - | - | - | - | - | - | 0.0% (0/28) | 0.0% (0/10) | 22.2% (4/18 |
| **15** | **Soft tissue abscess** | - | - | - | - | - | - | 0.0% (0/28) | 0.0% (0/10) | 27.8% (5/18) |

Supplementary table 5: Results of the mixed-model analysis according to confounders. Thoracic discs tended to have higher density than lumbar discs in 135kVp CT images and in cMaps. Mixed-model analysis revealed no effect of age on densities in our study population. Females tended to show higher IVD density than males in 135 kVp images and lower IVD density in cMaps.

*AD: Abnormal disc; cMaps:* Collagen-/chondroitin-sensitive map; *DECT: Dual-energy computed tomography; HU: Hounsfield unit; IVD: Intervertebral disc*

|  | **135 kVP CT images** | **DECT cMaps** |
| --- | --- | --- |
| **Spinal level of ADs** | 23.8 HU (95%CI 1.4 to 46.2), p=0.04) | 14.0 HU (95%CI -20.3 to 48.3), p=0.40 |
| **Patient age** | 0.1 HU (95% CI -0.4 to 0.6); p=0.65 | 0.1 HU (95% CI -0.6 to -0.9); p=0.76 |
| **Gender** | 4.3 HU (95%CI -13.6 to 22.3), p=0.62 | -1.9 HU (95%CI -29.4 to 25.6), p=0.88 |
